# Antimicrobial Efficacy of Aloe Vera, Chlorhexidine, Hyaluronic Acid, and Diode Laser Against Periodontopathogenic Bacteria: A Mixed-Methods Study

**DOI:** 10.7759/cureus.88283

**Published:** 2025-07-19

**Authors:** Ashwini Zadode, Lalitha Shiggaon, Samiksha Ghagre, Vaishali M Mane, Snehal B Chintale, Sakshi S Awaghad, Aarti Patil

**Affiliations:** 1 Department of Periodontics, Jawahar Medical Foundation's Annasaheb Chudaman Patil Memorial Dental College, Dhule, IND; 2 Department of Public Health Dentistry, Jawahar Medical Foundation's Annasaheb Chudaman Patil Memorial Dental College, Dhule, IND

**Keywords:** aloe vera gel, antibacterial agents, chlorhexidine gluconate, diode laser, hyaluronic acid, periodontitis

## Abstract

Introduction: This study aimed to assess and compare the antibacterial properties of aloe vera, chlorhexidine (CHX), hyaluronic acid (HA), and diode lasers against periodontal pathogens across various time intervals. The goals included evaluating the effectiveness of aloe vera, CHX, HA, and a diode laser at 0.5, 1, 2, and 5 minutes against periodontal pathogenic bacteria, with a focus on determining their antimicrobial efficacy.

Materials and methods: This mixed-methods study included 25 patients with chronic periodontitis (probing depth ≥ 5 mm at five or more sites with clinical attachment loss and ≥ 20 teeth). Subgingival plaque was collected from four quadrants per patient and randomized into four experimental groups (n = 25): 100% aloe vera gel (locally sourced, formulated with carboxymethylcellulose; Merck/MilliporeSigma, Burlington, Massachusetts, United States), 0.2% CHX mouthwash (Corsodyl; GSK plc, London, United Kingdom), 10% dilution of 0.8% high-molecular-weight HA (1.5 MDa) (Gengigel®; Ricerfarma S.r.l., Milan, Italy), and a 980 nm diode laser (Picasso; AMD Lasers, Indianapolis, Indiana, United States). In the control group (n =25), subgingival plaque was randomly collected from any quadrant. Viable colonies were counted on blood agar plates (HiMedia Laboratories Private Limited, Thane, Maharashtra, India) after 48 hours of incubation. The saline control (0.9%) (Baxter India Pvt. Ltd., Gurgaon, India) was processed similarly. Data were log-transformed and analyzed using multivariate analysis of variance (MANOVA) and Tukey’s test (p < 0.05).

Results: Diode laser treatment demonstrated the highest antibacterial efficacy, achieving complete pathogen eradication within two minutes. CHX showed rapid and sustained reductions, followed by HA, with significant effects at longer exposure times. Aloe vera exhibited the lowest efficacy, with notable reductions observed after two minutes. Saline did not exhibit antimicrobial effects. Treatment, time, and their interactions significantly influenced bacterial reduction (p < 0.001).

Conclusion: The 980 nm diode laser exhibited superior antibacterial efficacy for acute periodontal interventions, followed by CHX, HA, and aloe vera. These findings support the tailored use of adjunctive therapies based on exposure time and clinical needs for chronic periodontitis management.

## Introduction

Periodontitis, an inflammatory condition of the tissues surrounding and supporting the teeth, originates from gingival tissue and, if untreated, progresses to deeper structures, disrupting bone homeostasis and leading to tooth loss [1]. This multifactorial disease is primarily driven by bacterial biofilms, which are complex microbial communities on tooth surfaces [2]. Biofilms comprise both commensal and pathogenic bacteria, with dysbiosis, a shift toward pathogenic dominance due to factors such as poor oral hygiene or systemic conditions, and fueling disease progression [3]. This microbial shift transitions the oral microbiota from predominantly gram-positive aerobes to gram-negative anaerobes, with key periodontal pathogens such as *Porphyromonas gingivalis*, *Tannerella forsythia*, and *Treponema denticola* implicated in disease etiology [4]. Approximately 700 microbial species inhabit the oral cavity, mostly commensals. However, pathogenic subsets drive periodontitis [5].

The primary goal of periodontal therapy is to restore gingival health and preserve supporting tissues through non-surgical interventions, such as scaling and root planing (SRP), which mechanically removes plaque, calculus, and diseased tissue [6]. Adjunctive chemical agents, such as mouthwashes, enhance plaque control by altering the periodontal pocket ecosystem and reducing the number of pathogenic bacteria [7]. Chlorhexidine (CHX), a broad-spectrum antimicrobial, is the gold standard adjunct, effective at 0.12-0.2%, with low toxicity and no reported carcinogenic risk [8]. Studies have demonstrated that CHX mouthwash combined with SRP significantly reduces probing depth and improves clinical attachment levels by reshaping the microbiota composition [9,10]. However, drawbacks of CHX, including tooth discoloration and emerging antimicrobial resistance, have spurred interest in natural alternatives such as hyaluronic acid (HA), aloe vera, and diode laser therapy [11,12].

HA, a high-molecular-weight glycosaminoglycan, exhibits anti-inflammatory, anti-edematous, and antibacterial properties and modulates periodontal biofilm and immune responses [12,13]. Studies have reported HA’s growth-inhibitory effects on gram-negative bacteria, including *P. gingivalis*, at concentrations of 4 mg/mL [14]. Aloe vera, a *Liliaceae* plant, contains bioactive compounds (such as aloesin and anthraquinones) with immunomodulatory, anti-inflammatory, and antibacterial properties [15]. When used subgingivally with SRP, aloe vera significantly improved severe periodontitis outcomes [16]. Diode lasers (980 nm) offer promising disinfection, reducing specific pathogens such as *P. gingivalis* in chronic periodontitis [17]. Despite their efficacy, the heterogeneity in study design and pathogen-specific outcomes necessitates a comparative evaluation.

The present study was designed to analyze and compare the antimicrobial activity of aloe vera, CHX, HA, and diode laser treatment against periodontal pathogens over varying in vitro exposure times (0.5, 1, 2, and 5 minutes). The key focus was to determine their effectiveness in inhibiting periodontal pathogenic bacteria.

## Materials and methods

Study design and setting

This mixed-methods study involved in vivo and in vitro designs and was conducted at the Department of Periodontics, Jawahar Medical Foundation's Annasaheb Chudaman Patil Memorial Dental College, Dhule, India, from June 2023 to May 2024. This study adhered to the guidelines of the Declaration of Helsinki and good clinical practice. Ethical approval was granted by the Institutional Ethical Clearance Committee (IEC/NEW/INST/2022/2959/2022/17D). All patients provided written informed consent after being informed of the study objectives, procedures, and potential risks.

Sample size estimation

The sample size was calculated using G*Power software version 3.1.9.2 (Heinrich Heine University, Düsseldorf, Germany) with an estimated effect size of 0.33, based on a previous study comparing colony-forming units/mL (CFU/mL) between aloe vera and CHX [18]. Assuming an alpha error of 0.5% and statistical power of 80%, a minimum of 125 samples was required for the study. The study comprised five groups, with 25 samples per group. These plaque samples were obtained from 25 patients, with each patient contributing five specimens: one from each of the four quadrants in the experimental group and one randomly selected from any quadrant in the control group.

Eligibility criteria

Patients were included if they were diagnosed with chronic periodontitis according to the 2018 American Academy of Periodontology classification [19], aged ≥ 30 years, dentate with at least 20 natural teeth, and exhibited probing depth (PD) ≥ 5 mm at five or more sites with clinical attachment loss (CAL). The exclusion criteria were as follows: current smokers, chronic alcoholics, use of antibiotics within three months or any medication within 48 hours prior to sample collection, systemic conditions affecting periodontal health (such as diabetes and immunosuppression), pregnancy, lactation, or allergies to study agents.

Methodology

PD and CAL were measured using an UNC-15 periodontal probe (Hu-Friedy Mfg. Co., LLC, Chicago, Illinois, United States). PD was recorded from the gingival margin to the pocket base at 10-20 g to ensure probe alignment with the tooth contours. CAL was assessed at six sites per tooth (mesiobuccal, buccal, distobuccal, mesiolingual, lingual, and distolingual) by measuring the distance from the free gingival margin (FGM) to the cementoenamel junction (CEJ), and the distance from the FGM to the pocket base. CAL was calculated by subtracting FGM-CEJ from the FGM-pocket base when the FGM was coronal to the CEJ, or by adding them when the FGM was apical to the CEJ.

Aloe vera gel was prepared at a 100% concentration using locally sourced *Aloe barbadensis* leaves. The leaves were washed with water and 0.1% sodium hypochlorite (Merck/MilliporeSigma, Burlington, Massachusetts, United States), and the inner gel was then homogenized. Gels were formulated with 1-4% carboxymethylcellulose sodium (CMC) (Merck/MilliporeSigma) as a gelling agent and sodium metabisulfite, methyl paraben sodium, and propyl paraben sodium (Merck/MilliporeSigma) as preservatives. The gel was stored at 4 °C until the time of use [16].

The 0.2% CHX mouthwash was sourced from Corsodyl (GSK plc, London, United Kingdom) [20]. A 10% dilution of 0.8% high-molecular-weight HA (1.5 MDa) (Gengigel®, Ricerfarma S.r.l., Milan, Italy) was prepared and subsequently crosslinked using methacrylic anhydride (Merck/MilliporeSigma) under alkaline conditions (pH 8-10) [12]. A diode laser (Picasso, 980 nm; AMD Lasers, Indianapolis, Indiana, United States) was used. Laser therapy was performed using an optical fiber with a diameter of 200 μm in continuous wave (CW) mode with a 1 W power output. The power density of the fiber tip was 3184.7 W/cm^2^, and the fluency was 640 J/cm^2^ [21].

Microbiological media included Robertson’s Cooked Meat (RCM) medium and blood agar plates (HiMedia Laboratories Private Limited, Thane, Maharashtra, India). Additional supplies comprised 0.22% tapered absorbent paper points (Dentsply Sirona Inc., Charlotte, North Carolina, United States) and 0.9% saline (Baxter India Pvt. Ltd., Gurgaon, India).

Subgingival plaque was collected from periodontal pockets (≥ 5 mm) of four quadrants from each patient and randomly divided into four experimental groups using sterile curettes (Hu-Friedy Mfg. Co.) under aseptic conditions. The samples were transferred to RCM medium in an airtight anaerobic jar and transported to the microbiology laboratory within 2 hours. Periodontopathogens (*P. gingivalis, Aggregatibacter actinomycetemcomitans, Streptococcus viridans, T. forsythia, Fusobacterium nucleatum*) were identified using the Vitek 2 Compact System (bioMérieux SA, Marcy-l'Étoile, France).

Bacterial suspensions (10^6^ CFU/mL) were prepared from the identified pathogens. A 0.1 mL bacterial suspension was added to 10 mL of each agent and incubated at 37 °C for 0.5, 1, 2, or 5 minutes. Subsequently, 0.1 mL from each dilution was inoculated onto blood agar plates and incubated at 37°C for 48 hours. Bacterial colonies were enumerated using a manual colony counter and expressed as CFU/mL. The saline control was processed identically. For the laser group, a 0.1 mL bacterial suspension was inoculated onto blood agar plates and incubated at 37 °C for 48 hours. The plates were exposed to a 980 nm diode laser for 0.5, 1, 2, and 5 minutes. After irradiation, the colonies were subcultured under identical conditions for 48 hours, and viable counts were determined as CFU/mL.

Calibration and reliability

Clinical measurements were performed by a single, calibrated examiner. Intra-examiner reliability was assessed using the intraclass correlation coefficient (ICC), with 10 patients measured twice, one week apart, yielding ICC values of 0.92 for PD and 0.90 for CAL. Laboratory procedures were standardized, and duplicate samples were processed to ensure reproducibility (ICC > 0.95 for colony counts).

Statistical analysis

Data were analyzed using the IBM SPSS Statistics for Windows, version 25 (Released 2017; IBM Corp., Armonk, New York, United States). CFU data were log-transformed and assessed for normality using the Kolmogorov-Smirnov test, which confirmed a normal distribution. To compare CFUs across the four study groups and over multiple time intervals, multivariate analysis of variance (MANOVA) was performed, followed by post-hoc Tukey’s tests for pairwise comparisons. The significance threshold was set at p < 0.05.

## Results

**Figure 1:**
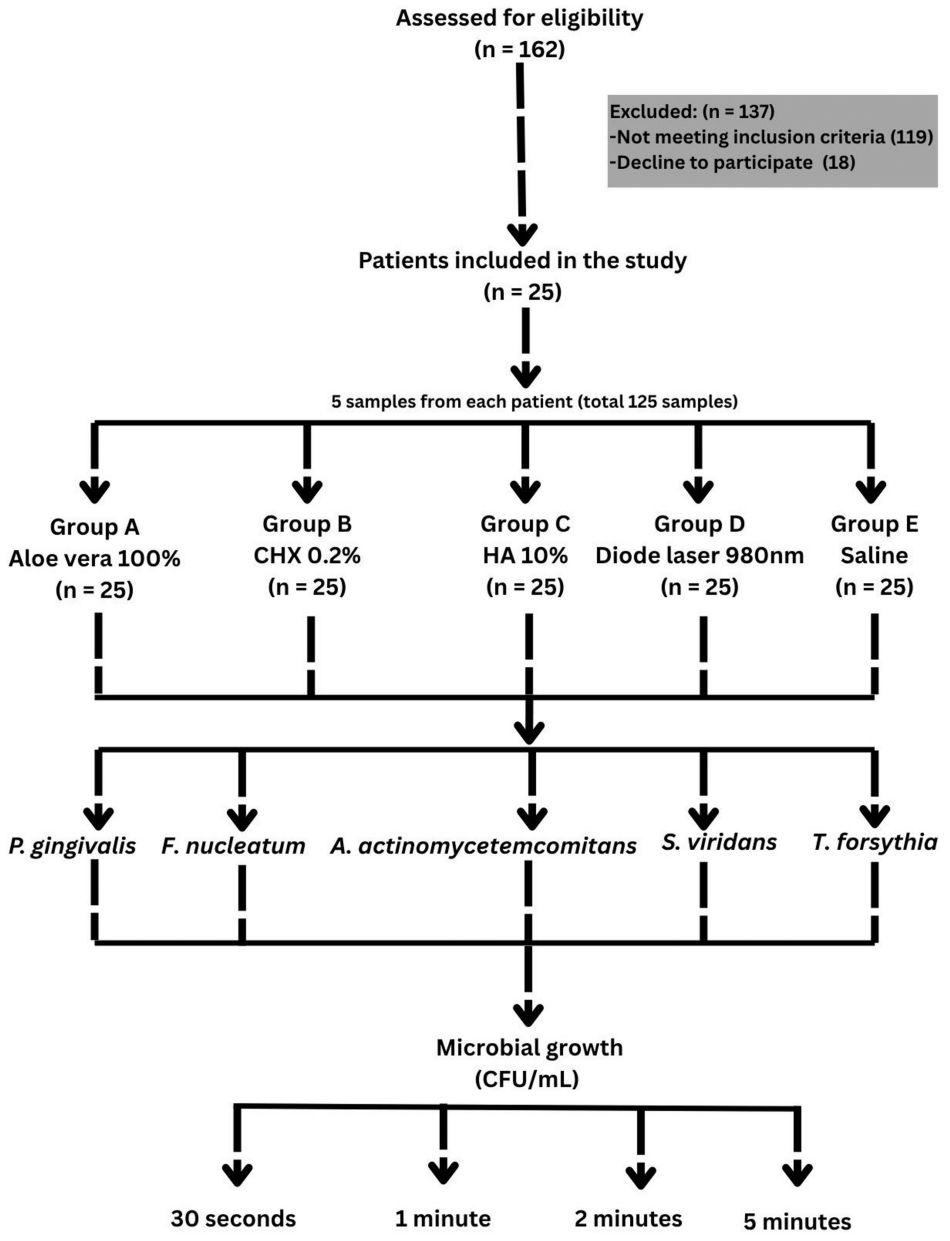
Flowchart of study design. CHX: chlorhexidine; HA: hyaluronic acid; *P. gingivalis*: *Porphyromonas gingivalis*; *A. actinomycetemcomitans*: *Aggregatibacter actinomycetemcomitans*; *S. viridans*: *Streptococcus viridans*; *T. forsythia*: *Tannerella forsythia*; *F. nucleatum*: *Fusobacterium nucleatum*; CFU: colony-forming units.

The flow diagram for this study is shown in Figure 1.

Of the 162 initially screened patients, 25 were eligible and included in the final study, with 16 (64%) male and nine (36%) female patients. The mean age was 42.50 ± 6.34 years for male patients and 38.45 ± 8.56 years for female patients. The PD averaged 6.20 ± 1.60 mm in male patients and 5.80 ± 1.20 mm in female patients, with no statistically significant differences observed (p > 0.05), as shown in Table 1.

**Table 1:** Descriptive analysis of study sample. *p-value > 0.05: non-significant. Chi-square goodness-of fit test was used for participants (test statistic χ²-value); Independent t test was used for age and probing depth (test statistics t-value).

Parameters	Sex		Test statistics	p-value
	Male	Female		
Participants, n (%)	16 (64.0)	9 (36.0)	1.96	0.162*
Age (years), mean ± SD	42.50 ± 6.34	38.45 ± 8.56	1.90	0.061*
Probing depth (mm), mean ± SD	6.20 ± 1.60	5.80 ± 1.20	1.01	0.322*

The mean log-transformed CFU/mL values revealed a significant variation in bacterial reduction across the treatment groups. For *P. gingivalis*, the lowest mean CFU was observed with laser treatment, followed by HA gel and aloe vera gel, whereas CHX showed moderate efficacy. The saline control group exhibited the highest bacterial load. A similar trend was observed in *A. actinomycetemcomitans, S. viridans, T. forsythia, *and *F. nucleatum*. These findings suggested that laser therapy and HA were more effective in reducing periodontal pathogens than CHX and aloe vera, with saline confirming the baseline bacterial proliferation (Table 2).

**Table 2:** Inferential analysis of CFU/ml as CFU/mL (log-transformed values) for bacterial species in study groups (N = 25) CHX: chlorhexidine; HA: hyaluronic acid; CFU: colony-forming unit

Groups	Porphyromonas gingivalis	Streptococcus viridans	Aggregatibacter actinomycetemcomitans	Fusobacterium nucleatum	Tannerella forsythia
	Mean ± SD	Mean ± SD	Mean ± SD	Mean ± SD	Mean ± SD
Aloe vera (100%)	2.88 ± 2.79	2.46 ± 2.6	3.35 ± 3.23	2.83 ± 2.71	3.06 ± 2.90
CHX (0.2%)	4.27 ± 1.41	3.87 ± 1.50	5.12 ± 1.80	4.16 ± 1.49	4.26 ± 1.67
HA (10%)	1.73 ± 1.61	1.55 ± 1.51	2.12 ± 1.70	1.68 ± 1.58	1.78 ± 1.63
Diode laser (980 nm)	1.22 ± 1.26	1.08 ± 1.10	1.41 ± 1.45	1.12 ± 1.14	1.26 ± 1.29
Saline	8.00 ± 0.00	8.00 ± 0.00	8.00 ± 0.00	8.00 ± 0.00	8.00 ± 0.00

Inferential analysis of CFU/mL (log-transformed values) for bacterial species at different time points revealed significant variations among the treatment groups. Aloe vera gel showed a gradual reduction in bacterial counts over time, with the most notable decline occurring between one and five minutes for all species, particularly *P. gingivalis* (6.30 to 3.70) and *T. forsythia* (6.30 to 3.70). CHX demonstrated rapid antimicrobial activity, with bacterial counts decreasing sharply within 0.5 minutes and continuing to decline, reaching their lowest levels at five minutes. HA also exhibited a significant reduction, with the most pronounced effect observed at five minutes. The 980 nm laser group showed the most drastic reduction, with complete elimination of all bacterial species within two minutes. In contrast, the saline control group maintained a consistently high bacterial count, confirming the absence of an antimicrobial effect. These findings suggested that although aloe vera, CHX, and HA were effective, the 980 nm laser was the most potent, achieving complete bacterial eradication within two minutes (Table 3).

**Table 3:** Inferential analysis of CFU/mL as CFU/mL (log-transformed values) for bacterial species at different time points CHX: chlorhexidine, HA: hyaluronic acid; CFU: colony-forming unit

Groups	Time	Porphyromonas gingivalis	Streptococcus viridans	Aggregatibacter actinomycetemcomitans	Fusobacterium nucleatum	Tannerella forsythia
		Mean ± SD	Mean ± SD	Mean ± SD	Mean ± SD	Mean ± SD
Aloe vera (100%)	0.5 min	6.30 ± 0.54	5.30 ± 0.68	6.48 ± 0.68	5.30 ± 0.89	6.30 ± 0.68
	1 min	6.00 ± 0.23	5.00 ± 0.45	6.30 ± 0.42	5.00 ± 0.67	6.00 ± 0.78
	2 min	4.00 ± 0.14	4.00 ± 0.52	4.30 ± 0.34	4.70 ± 0.48	5.00 ± 0.48
	5 min	3.70 ± 0.26	3.00 ± 0.56	4.00 ± 0.23	3.30 ± 0.58	3.70 ± 0.56
CHX (0.2%)	0.5 min	3.70 ± 0.18	3.60 ± 0.76	4.00 ± 0.65	3.74 ± 0.78	3.78 ± 0.78
	1 min	3.30 ± 0.45	3.18 ± 0.45	3.78 ± 0.76	3.30 ± 0.64	3.04 ± 0.54
	2 min	2.95 ± 0.54	2.85 ± 0.58	3.60 ± 0.67	3.00 ± 0.65	2.97 ± 0.56
	5 min	2.30 ± 0.34	2.00 ± 0.21	3.00 ± 0.56	2.18 ± 0.78	2.37 ± 0.28
HA (10%)	0.5 min	3.70 ± 0.67	3.40 ± 0.45	3.95 ± 0.34	3.60 ± 0.72	3.74 ± 0.24
	1 min	2.95 ± 0.56	2.78 ± 0.56	3.70 ± 0.56	2.90 ± 0.56	3.00 ± 0.45
	2 min	2.60 ± 0.54	2.30 ± 0.62	3.00 ± 0.23	2.48 ± 0.34	2.70 ± 0.34
	5 min	1.48 ± 0.21	1.00 ± 0.16	2.00 ± 0.34	1.40 ± 0.23	1.60 ± 0.12
Diode laser (980 nm)	0.5 min	2.60 ± 0.34	2.30 ± 0.34	2.95 ± 0.43	2.30 ± 0.34	2.65 ± 0.21
	1 min	2.30 ± 0.23	2.00 ± 0.32	2.70 ± 0.56	2.18 ± 0.43	2.40 ± 0.32
	2 min	0.00 ± 0.00	0.00 ± 0.00	0.00 ± 0.00	0.00 ± 0.00	0.00 ± 0.00
	5 min	0.00 ± 0.00	0.00 ± 0.00	0.00 ± 0.00	0.00 ± 0.00	0.00 ± 0.00
Saline	0.5 min	8.00 ± 2.34	8.00 ± 2.34	8.00 ± 2.34	8.00 ± 2.34	8.00 ± 2.34
	1 min	8.00 ± 2.34	8.00 ± 2.34	8.00 ± 2.34	8.00 ± 2.34	8.00 ± 2.34
	2 min	8.00 ± 2.34	8.00 ± 2.34	8.00 ± 2.34	8.00 ± 2.34	8.00 ± 2.34
	5 min	8.00 ± 2.34	8.00 ± 2.34	8.00 ± 2.34	8.00 ± 2.34	8.00 ± 2.34

The MANOVA test revealed significant effects of treatment (group), time, and their interaction on CFU/mL (log-transformed). Pillai’s trace showed a strong treatment effect (p < 0.001), indicating significant differences among the aloe vera, CHX, HA, diode laser, and saline groups. Time also had a substantial effect (p < 0.001), confirming the bacterial reduction over time. The Group × time interaction was significant (p < 0.001), suggesting that the treatment efficacy varied by duration (Table 4).

**Table 4:** Comparison of groups using multivariate analysis of variance (MANOVA) test. Pillai trace value (value ranges from 0 to 1, where 1 represent the stronger evidence); *p-value < 0.05: significant

Effect	Pillai trace value	F-value	p-value
Treatment (Group)	0.82	25.67	0.001*
Time	0.95	40.45	0.001*
Group × Time Interaction	0.52	15.78	0.001*

A time-specific MANOVA (Pillai’s trace) analysis revealed significant differences in treatment efficacy (p < 0.05). The 980 nm laser showed the highest antimicrobial effect at all time points, followed by CHX 0.2% and HA 10%. Aloe vera 100% exhibited delayed but increasing efficacy, becoming significant only after two minutes. Saline had no effect on any of the intervals. These results confirmed that laser therapy was the fastest and most potent, whereas CHX and HA were progressively effective, and aloe vera required longer exposure for significant bacterial reduction (Table 5).

**Table 5:** Time-specific treatment efficacy using multivariate analysis of variance (MANOVA) test *Pillai trace value (value ranges from 0 to 1, where 1 represent the stronger evidence). CHX: chlorhexidine; HA: hyaluronic acid

Time	Diode laser 980 nm	CHX 0.2%	HA 10%	Aloe vera 100%	Saline
0.5 min	0.92*	0.65*	0.58*	0.12	0
1 min	0.95*	0.72*	0.63*	0.18	0
2 min	1.00*	0.80*	0.70*	0.45*	0
5 min	1.00*	0.85*	0.78*	0.52*	0

The bacterium-specific MANOVA (Pillai’s Trace) results demonstrated significant antimicrobial efficacy (p < 0.05) across all treatments except saline (0). The 980 nm laser achieved complete eradication (1.00) for all bacterial species, confirming its superior broad-spectrum activity. CHX showed strong effectiveness (0.75-0.82), with the greatest effect on *P. gingivalis* (0.82). HA exhibited moderate efficacy (0.65-0.75), whereas aloe vera had the weakest but still significant effect (0.38-0.48). These findings highlighted that diode laser therapy was universally potent, CHX and HA were variably effective, and aloe vera had limited but measurable antibacterial action against all tested pathogens (Table 6).

**Table 6:** Bacterium-specific differences analyzed using multivariate analysis of variance (MANOVA) *Pillai trace value (value ranges from 0 to 1, where 1 represent the stronger evidence). CHX: chlorhexidine; HA: hyaluronic acid

Bacteria	Diode laser 980 nm	CHX 0.2%	HA 10%	Aloe vera 100%	Saline
Porphyromonas gingivalis	1.00*	0.82*	0.75*	0.48*	0
Streptococcus viridans	1.00*	0.78*	0.70*	0.42*	0
Aggregatibacter actinomycetemcomitans	1.00*	0.75*	0.65*	0.38*	0
Fusobacterium nucleatum	1.00*	0.80*	0.72*	0.45*	0
Tannerella forsythia	1.00*	0.77*	0.68*	0.40*	0

The post-hoc Tukey’s test revealed significant differences (p < 0.05) in antimicrobial efficacy among all treatment groups. The 980 nm laser demonstrated superior performance compared to CHX, HA, aloe vera, and saline. CHX was significantly more effective than HA and aloe vera; however, HA outperformed aloe vera. All active treatments showed significantly greater efficacy than saline (p < 0.001), confirming their antimicrobial potential in a hierarchical order: diode laser > CHX > HA > aloe vera (Table 7).

**Table 7:** Pairwise comparison of groups with post-hoc Tukey’s test Pillai trace value (value ranges from 0 to 1, where 1 represent the stronger evidence); *p-value < 0.05: significant CHX: chlorhexidine; HA: hyaluronic acid

Comparison	Pillai's Trace	p-value	Interpretation
Diode laser vs. CHX	0.65	0.001*	Diode laser superior to CHX
Diode laser vs. HA	0.78	0.001*	Diode laser superior to HA
Diode laser vs. aloe vera	0.82	0.001*	Diode lase superior to aloe vera
Diode laser vs. saline	0.98	0.001*	Diode lase superior to saline
CHX vs. HA	0.28	0.003*	CHX superior to HA
CHX vs. aloe vera	0.53	0.001*	CHX superior to aloe vera
CHX vs. saline	0.85	0.001*	CHX superior to saline
HA vs. aloe vera	0.32	0.001*	HA superior to aloe vera
HA vs. saline	0.70	0.001*	HA superior to saline
Aloe vera vs. saline	0.75	0.001*	Aloe vera superior to saline

## Discussion

The findings of this study provided valuable insights into the comparative antibacterial efficacy of various adjunctive therapies, including aloe vera, CHX, HA, and diode laser, used in conjunction with SRP for the management of chronic periodontitis. The results highlighted the superior antimicrobial performance of the 980 nm diode laser, followed by CHX and HA, with aloe vera demonstrating the least but still significant antibacterial activity.

The diode laser’s ability to achieve complete bacterial eradication within a short exposure time underscores its potential as a highly effective adjunctive therapy. This outcome is consistent with the mechanism of laser therapy, which employs photothermal and photochemical effects to disrupt bacterial cell walls and inhibit microbial proliferation [22]. The high power density and fluency of the 980 nm diode laser likely contributed to its rapid bactericidal action, targeting both gram-negative and gram-positive periodontal pathogens. In support of this, Qadri et al. conducted a systematic review encompassing 10 trials and concluded that the application of diode lasers in conjunction with SRP yielded superior outcomes for patients with periodontitis and probing pocket depths ≤ 5 mm as opposed to the use of SRP in isolation [23]. They observed that diminished efficacy and, in certain instances, even inferior results within the diode laser treatment cohorts were prevalent in studies utilizing 810 nm diode lasers.

Samulak et al. reported a statistically significant reduction in *P. gingivalis, T. denticola, T. forsythia, Micromonas micros, *and* Eubacterium nodatum* after three months of treatment with a 980 nm diode laser [21]. The rapid action observed in our study, with complete bacterial elimination within two minutes, further corroborates the findings of Kamma et al., who reported that diode lasers effectively sterilized periodontal pockets by targeting specific pathogens without causing significant thermal damage to surrounding tissues in cases of aggressive periodontitis during the six-month observation period [24].

However, the superior performance of the diode laser contrasts with some studies that have reported limited long-term benefits. For instance, Slot et al. conducted a systematic review and found that while diode lasers provide immediate bacterial reduction, their long-term clinical benefits over SRP alone remain inconclusive owing to the variability in study protocols and laser parameters [25]. This discrepancy suggests that while the diode laser excels in in vitro and short-term in vivo settings, its clinical efficacy may depend on standardized application protocols and patient-specific factors such as pocket depth and systemic health.

CHX’s rapid antimicrobial activity observed within the initial exposure period reaffirms its status as the gold standard for periodontal therapy. Its broad-spectrum activity, attributed to its cationic nature, disrupts bacterial cell membranes, leading to immediate reductions in the microbial load. This aligns with the findings of Poppolo and Ouanounou, who reported that 0.2% CHX mouthwash, when used adjunctively with SRP, significantly reduced periodontal pathogens and improved clinical outcomes such as PD and CAL [20]. The present study’s results, showing CHX’s consistent efficacy across multiple pathogens, particularly *P. gingivalis*, are supported by its established ability to adsorb onto oral surfaces, providing a sustained antimicrobial effect [10].

Despite its efficacy, CHX’s limitations, such as tooth discoloration and potential for antimicrobial resistance, warrant consideration. A study by Zanataa et al. highlighted patient dissatisfaction with CHX due to aesthetic concerns, particularly staining with 0.12% CHX, which may affect compliance [11]. Additionally, recent concerns regarding emerging resistance, as discussed by Abbood et al., suggest that prolonged CHX use could select for resistant bacterial strains, potentially diminishing its long-term efficacy [26]. These drawbacks underscore the need for alternative agents to balance the efficacy and patient acceptability, as explored in this study.

HA showed a moderate but significant antibacterial effect, particularly at longer exposure times, making it a viable natural alternative to CHX. Its anti-inflammatory and antibacterial properties, likely mediated by its ability to modulate the periodontal microenvironment, are consistent with the findings of previous studies [12-14]. The current study’s observation of HA’s efficacy against gram-negative bacteria, such as *P. gingivalis*, is consistent with Pirnazar et al., who demonstrated HA’s growth-inhibitory effects on periodontal pathogens at specific concentrations [13]. HA’s biocompatibility and the lack of reported adverse effects, such as staining, make it an attractive option for patients concerned about CHX’s side effects.

However, HA’s delayed onset of action compared to CHX and diode laser therapy suggests a different mechanism, possibly involving immune modulation rather than direct bactericidal activity. This is supported by a previous review, which found that HA’s primary benefit lies in its ability to reduce inflammatory markers rather than directly targeting bacterial viability [14]. This contrast indicates that HA may be better suited for long-term maintenance therapy than for immediate bacterial eradication, highlighting the need for further studies to optimize its concentration and delivery for maximum antimicrobial impact.

The antibacterial activity of aloe vera, although less potent than that of other agents, still contributed to significant reductions in bacterial counts, particularly with prolonged exposure. This aligns with known bioactive compounds, such as anthraquinones and aloesin, which exhibit antibacterial and anti-inflammatory properties [15]. Ashouri et al. found that subgingival aloe vera gel application improved periodontal parameters in patients with chronic periodontitis, supporting its role as an adjunctive therapy [16]. The delayed efficacy observed in the current study may reflect aloe vera’s reliance on cumulative exposure to achieve antimicrobial effects, as suggested by Jain et al., who noted its effectiveness against *A. actinomycetemcomitans, Clostridium bacilli, Streptcoccus mutans, *and* Staphlococcus aureus* with 100% aloe vera gel, compared to 50% aloe vera gel [27].

In contrast, the weaker performance of aloe vera compared to CHX, HA, and diode laser therapy may be attributed to its variable bioactive content and the lack of standardized formulations. Vangipuram et al. found similar efficacy of aloe vera mouthwash and 0.12% CHX in periodontitis [18], highlighting inconsistencies in aloe vera efficacy due to differences in gel preparation and concentration, which could explain the less pronounced effect observed here. These findings suggest that while aloe vera holds promise as a natural, cost-effective option, its clinical application may require optimized formulations to enhance its antimicrobial potency.

The findings of this study regarding time-dependent antibacterial efficacy suggest that mouthwash exposure duration significantly influences periodontal pathogen reduction. For optimal results, 0.2% CHX mouthwash should be used for 30-60 seconds twice daily, as its rapid antimicrobial action is effective within short intervals. The HA mouthwash, which requires longer exposure, is best used for one to two minutes twice daily to maximize antibacterial and anti-inflammatory effects. A 100% aloe vera mouthwash with delayed efficacy should be used for two to five minutes twice daily to achieve significant bacterial reduction. These durations align with clinical recommendations for balancing efficacy and patient compliance in periodontal therapy [7,8,14,15].

Clinical implications

The hierarchical order of efficacy, diode laser > CHX > HA > aloe vera, reflects the varying mechanisms and clinical applicability of these agents. The diode laser’s rapid, broad-spectrum activity makes it ideal for acute interventions, particularly in deep periodontal pockets where mechanical debridement alone may be insufficient. CHX remains a reliable choice for immediate and sustained antimicrobial effects; however, careful consideration of its side effects is required. As natural alternatives, HA and aloe vera offer biocompatible options with fewer adverse effects; however, their slow action may limit their use in acute settings.

The significant interaction between treatment and time suggests that the exposure duration is a critical factor in optimizing antimicrobial outcomes. This is particularly relevant for aloe vera and HA, which showed increasing efficacy over time, indicating their potential for use in the maintenance phase of periodontal therapy. Bacterium-specific analysis further highlights the need for tailored approaches, as pathogens such as *P. gingivalis* respond differently across treatments, with diode laser and CHX showing superior activity.

Limitations and future directions

While this study provides robust comparative data, its limitations include the in vitro nature of some experiments, which may not fully replicate the complex in vivo periodontal environment. Additionally, a focus on specific pathogens may overlook broader microbial community dynamics in periodontitis. Future research should explore long-term clinical outcomes, including patient-centered metrics, such as comfort and compliance, and investigate standardized formulations for natural agents, such as aloe vera, to enhance reproducibility. Combination therapies, such as HA with diode laser or aloe vera with CHX, can also be evaluated using well-designed randomized controlled trials to leverage synergistic effects.

## Conclusions

This study demonstrated that the 980 nm diode laser exhibited the highest antimicrobial efficacy among the tested adjunctive therapies, achieving complete bacterial eradication within a short exposure time. CHX is a highly effective agent with rapid and sustained antibacterial activity, reinforcing its role as the gold standard for periodontal therapy. HA showed moderate but significant antibacterial effects, particularly with prolonged exposure, offering a biocompatible alternative with potential for long-term use. Aloe vera, while effective, displayed the least potency and required longer exposure to achieve measurable bacterial reduction. The hierarchical efficacy of these agents highlights the potential of diode laser therapy for acute interventions and the complementary roles of CHX, HA, and aloe vera in periodontal management. These findings underscore the importance of tailoring adjunctive therapies based on treatment goals, exposure duration, and patient-specific needs to optimize outcomes in chronic periodontitis management.

## References

[1] Bhuyan R, Bhuyan SK, Mohanty JN, Das S, Juliana N, Juliana IF (2022). Periodontitis and its inflammatory changes linked to various systemic diseases: a review of its underlying mechanisms. Biomedicines.

[2] Hajishengallis G (2015). Periodontitis: from microbial immune subversion to systemic inflammation. Nat Rev Immunol.

[3] Lamont RJ, Hajishengallis G (2015). Polymicrobial synergy and dysbiosis in inflammatory disease. Trends Mol Med.

[4] Marsh PD (2006). Dental plaque as a biofilm and a microbial community - implications for health and disease. BMC Oral Health.

[5] Avula H, Chakravarthy Y (2022). Models of periodontal disease pathogenesis: a journey through time. J Indian Soc Periodontol.

[6] Kwon T, Lamster IB, Levin L (2021). Current concepts in the management of periodontitis. Int Dent J.

[7] Vyas T, Bhatt G, Gaur A, Sharma C, Sharma A, Nagi R (2021). Chemical plaque control - a brief review. J Family Med Prim Care.

[8] Brookes ZL, Bescos R, Belfield LA, Ali K, Roberts A (2020). Current uses of chlorhexidine for management of oral disease: a narrative review. J Dent.

[9] James P, Worthington HV, Parnell C (2017). Chlorhexidine mouthrinse as an adjunctive treatment for gingival health. Cochrane Database Syst Rev.

[10] Bescos R, Ashworth A, Cutler C (2020). Effects of chlorhexidine mouthwash on the oral microbiome. Sci Rep.

[11] Zanatta FB, Antoniazzi RP, Rösing CK (2010). Staining and calculus formation after 0.12% chlorhexidine rinses in plaque-free and plaque covered surfaces: a randomized trial. J Appl Oral Sci.

[12] Jentsch H, Pomowski R, Kundt G, Göcke R (2003). Treatment of gingivitis with hyaluronan. J Clin Periodontol.

[13] Pirnazar P, Wolinsky L, Nachnani S, Haake S, Pilloni A, Bernard GW (1999). Bacteriostatic effects of hyaluronic acid. J Periodontol.

[14] Al-Shammari NM, Shafshak SM, Ali MS (2018). Effect of 0.8% hyaluronic acid in conventional treatment of moderate to severe chronic periodontitis. J Contemp Dent Pract.

[15] Grindlay D, Reynolds T (1986). The Aloe vera phenomenon: a review of the properties and modern uses of the leaf parenchyma gel. J Ethnopharmacol.

[16] Ashouri Moghaddam A, Radafshar G, Jahandideh Y, Kakaei N (2017). Clinical evaluation of effects of local application of aloe vera gel as an adjunct to scaling and root planning in patients with chronic periodontitis. J Dent (Shiraz).

[17] Verma V, Ojha M, Yadav S, Ranjan M, Hassan S, Dhull KS (2023). Effectiveness of diode lasers in the reduction of bacteremia associated with ultrasonic scaling: a clinical and microbiological study. J Pharm Bioallied Sci.

[18] Vangipuram S, Jha A, Bhashyam M (2016). Comparative efficacy of aloe vera mouthwash and chlorhexidine on periodontal health: a randomized controlled trial. J Clin Exp Dent.

[19] Caton JG, Armitage G, Berglundh T (2018). A new classification scheme for periodontal and peri-implant diseases and conditions - introduction and key changes from the 1999 classification. J Clin Periodontol.

[20] Poppolo Deus F, Ouanounou A (2022). Chlorhexidine in dentistry: pharmacology, uses, and adverse effects. Int Dent J.

[21] Samulak R, Suwała M, Dembowska E (2021). Nonsurgical periodontal therapy with/without 980 nm diode laser in patients after myocardial infarction: a randomized clinical trial. Lasers Med Sci.

[22] Elavarasu S, Naveen D, Thangavelu A (2012). Lasers in periodontics. J Pharm Bioallied Sci.

[23] Qadri T, Javed F, Johannsen G, Gustafsson A (2015). Role of diode lasers (800-980 nm) as adjuncts to scaling and root planing in the treatment of chronic periodontitis: a systematic review. Photomed Laser Surg.

[24] Kamma JJ, Vasdekis VG, Romanos GE (2009). The effect of diode laser (980 nm) treatment on aggressive periodontitis: evaluation of microbial and clinical parameters. Photomed Laser Surg.

[25] Slot DE, Jorritsma KH, Cobb CM, Van der Weijden FA (2014). The effect of the thermal diode laser (wavelength 808-980 nm) in non-surgical periodontal therapy: a systematic review and meta-analysis. J Clin Periodontol.

[26] Abbood HM, Hijazi K, Gould IM (2023). Chlorhexidine resistance or cross-resistance, that is the question. Antibiotics (Basel).

[27] Jain S, Rathod N, Nagi R (2016). Antibacterial effect of aloe vera gel against oral pathogens: an in-vitro study. J Clin Diagn Res.

